# Conjoint Analysis of SMRT- and Illumina-Based RNA-Sequencing Data of *Fenneropenaeus chinensis* Provides Insight Into Sex-Biased Expression Genes Involved in Sexual Dimorphism

**DOI:** 10.3389/fgene.2019.01175

**Published:** 2019-11-15

**Authors:** Qiong Wang, Yuying He, Jian Li

**Affiliations:** ^1^Key Laboratory for Sustainable Utilization of Marine Fisheries Resources, Ministry of Agriculture and Rural Affairs, Yellow Sea Fisheries Research Institute, Chinese Academy of Fishery Sciences, Qingdao, China; ^2^Function Laboratory for Marine Fisheries Science and Food Production Processes, Qingdao National Laboratory for Marine Science and Technology, Qingdao, China

**Keywords:** shrimp, full-length transcriptome, growth, body color, reproduction

## Abstract

*Fenneropenaeus chinensis* (*F. chinensis*) is one of the most commercially important cultured shrimps in China. The adult *F. chinensis* exhibit sexual dimorphism in growth and body color. In this research, we profiled the whole transcriptome of *F. chinensis* by using single molecule real-time-based full-length transcriptome sequencing. We further performed Illumina-based short reads RNA-seq on muscle and gonad of two sexes to detect the sex-biased expression genes. In muscle, we observed significantly more female-biased transcripts. With the differentially expressed transcripts (DETs) in muscle, some pathways related to the energy metabolism were enriched, which may be responsible for the difference of growth. We also digged out a pathway named porphyrin and chlorophyll metabolism. It was speculated to relevant to the difference of body color between the two sexes of shrimp. Interestingly, almost all DETs in these pathways were female-biased expression in muscle, which could explain the phenomenon of better growth performance and darker body color in female. In gonad, several pathways involved in reproduction were enriched. For instance, some female-biased DETs participated in the arachidonic acid metabolism, which was reported crucial in female reproduction. In conclusion, our studies identified abundant sex-biased expression transcripts and important pathways involved in sexual dimorphism by using the RNA-seq method. It provided a basis for future researches on the sexual dimorphism of *F. chinensis.*

## Introduction

*Fenneropenaeus chinensis* (*F. chinensis*), which belongs to the family *Penaeidae* of *Crustacea*, is one of the most commercially important cultured shrimps in China. It mainly distributes in the Yellow Sea and Bohai Sea of China and west and south coast of the Korean Peninsula (Wang et al., 2017). Due to the delicious taste and rich nutrition, the *F. chinensis* is becoming more and more popular in consumers.

There are many species exhibit pronounced sexual dimorphism in nature. They usually show different colors, shapes, or body weight in different sexes, like chicken, peacock, guppy, and so on. The extensive sexual dimorphism in nature accord with the Darwin’s conjecture that sexual selection is a force distinct from natural selection (Lande, 1980; Tipton, 1999). Sexual dimorphism is an extreme form of phenotypic plasticity. Studies on sexual dimorphism are significative for the wide intra-specific variations (Mank, 2017). In the process of cultivation, the color of adult female shrimps of *F. chinensis* tend to blue, while the males tend to yellow. The adult females were observed bigger in body size and heavier in body weight than males. Body weight is an important economical trait in production. Locate the genes related to growth will accelerate the process of molecular breeding for *F. chinensis*.

Gene expression plays an important role in generating the phenotypic diversity since there was limited genetic divergence in genome of organism. Most of sexual dimorphism are caused by the differential expression of genes between different sexes, which is known as sex-biased gene expression (Ellegren and Parsch, 2007; Grath and Parsch, 2016). Sex-biased genes could be classified as either male-biased or female-biased expression depending on which sex expresses higher (Grath and Parsch, 2016).

In recent years, short reads RNA sequencing (RNA-seq) technique has become an important tool in biological studies. It is powerful for uncovering the relationship between genotype and phenotype (Qian et al., 2014; Wang et al., 2018). However, the short reads, mostly 100∼300 bp, bring many challenges to the transcriptome assemble. For instance, it is difficult to identify the alternative splicing with short reads; the repetitive sequence also could cause confusion in the assemble. Recently, the third-generation sequencing technology has sharply increased the length of sequencing reads (Grabherr et al., 2011). The PacBio platform even could sequence the whole molecule of mRNA (Rhoads and Au, 2015). Due to the much longer reads length, the complex sequence such as repetitive regions could be displayed within a single read. It could achieve the full-length (FL) sequence of transcripts and identify full coding sequences and multiple encoded isoforms (Weirather et al., 2017).

The FL transcriptome sequencing technology has prompt the overall annotation of the transcriptome and the subsequent studies in many species, such as fission yeast (Kuang et al., 2017), zebrafish (Nudelman et al., 2018), and mouse (Tardaguila et al., 2018). Especially for the non-reference genome organisms, the FL transcriptome sequencing made it possible to fully characterize the novel transcript (Grabherr et al., 2011). For example, it provides insight into the adaptive divergent function in extreme metabolism of the ruby-throated hummingbird, a non-reference species (Workman et al., 2018). In aquaculture, this technology has been applied to some analysis of important characters. FL transcriptome sequencing on pacific abalone characterized the transcriptome information for female and male individuals, and identified some sex-specific isoforms (Kim et al., 2017). For Pacific white shrimp *Litopenaeus vannamei*, a species belongs to the same genus of *Penaeus* with *F. chinensis*, transcript expression profiles survey provided insight into the immune mechanism of shrimps (Zhang et al., 2019).

Due to the abundant of repetitive sequence and high heterozygosity (Gao and Kong, 2005; Wang et al., 2008), the genome of *F. chinensis* has not been completely sequenced yet. To make a reference in this research, we used a fast growth cultured breed of *F. chinensis*, “Huanghai No. 1,” which was raised by continuous selection of several generations, to profile the whole transcriptome of *F. chinensis*. We further performed short reads RNA-seq on muscle and gonad of two sexes of shrimps to detect the sex-biased expression genes.

## Results

### Expression Profiles Delineated by the Full-Length Transcriptome Sequencing

We obtained 24.81 Gb single molecule real-time (SMRT) clean data in total. Circular consensus (CCS) sequences were extracted from the original sequence according to the condition of full passes> = 1 and the sequence accuracy > 0.90. A total of 473,469 CCS reads were extracted (**Table 1**). Among them 382,500 were full length reads non-chimeric (FLNC). The FLNC sequences were clustered and we obtained 17,470 consensus isoforms with mean length of 2,191 (**Supplementary Figure S1**). After polished, 17,279 high-quality consensus isoforms and 190 low-quality consensus isoforms were obtained. The low-quality consensus isoforms were corrected with the Illumina RNA-seq data, and merged with the high-quality FL consensus isoforms. Isoforms with high identity (>0.99) were removed redundancy. Finally, we obtained 10,795 high-quality non-redundant FL transcripts with mean length of 2,315 bp. The completeness of the non-redundant FL transcripts were assessed by BUSCO (Simao et al., 2015), and result showed that the percent of the complete transcripts identified in our project was more than 65% (**Supplementary Figure S2**). These 10,795 non-redundant FL transcripts were regarded as reference transcriptome in the following analysis.

**Table 1:** Summary of full-length transcriptome sequencing production.

Type	Count
Circular consensus (CCS) reads	473,469
Full length reads non-chimeric (FLNC)	382,500
FL consensus isoforms	17,470
High-quality FL transcripts	17,279
Non-redundancy high-quality FL transcripts	10,795
Alternative splicing	162
Simple sequence repeats (SSR)	10,941
coding sequences (CDS)	8,231
lncRNA	823
Annotation	9,177

The precursor of mRNA (pre-mRNA) has a variety of splicing types. Different exons are selected to produce different mature mRNAs, which was called alternative splicing (AS). The FL sequences were pairwise compared, and 162 AS events were detected (**Supplementary Table S1**). Simple sequence repeats (SSR) are short (1∼6 bp) tandemly repeated DNA sequences. It is also known as microsatellites. In this study, we totally identified 10,941 SSR (**Supplementary Table S2**). Most of the them were mono-nucleotide repeats (**Supplementary Figure S3**). There were 10,238 coding sequences (CDS) predicted in all and 8,231 of them possessed complete open reading frames (ORF) (**Supplementary Table S3**). Four approaches (CPC/CNCI/CPAT/Pfam) were used to predicted long non-coding RNA (lncRNA), and 823 lncRNA were identified by all four methods consistently (**Supplementary Figure S4**). The function of 9,177 high quality FL transcripts were annotated by conjoint analysis of a series of annotation databases (**Supplementary Table S4**).

### Illumina-Based Ribonucleic Acid Sequencing Data Displayed the Expression Pattern of Each Transcript

**Figure 1:**
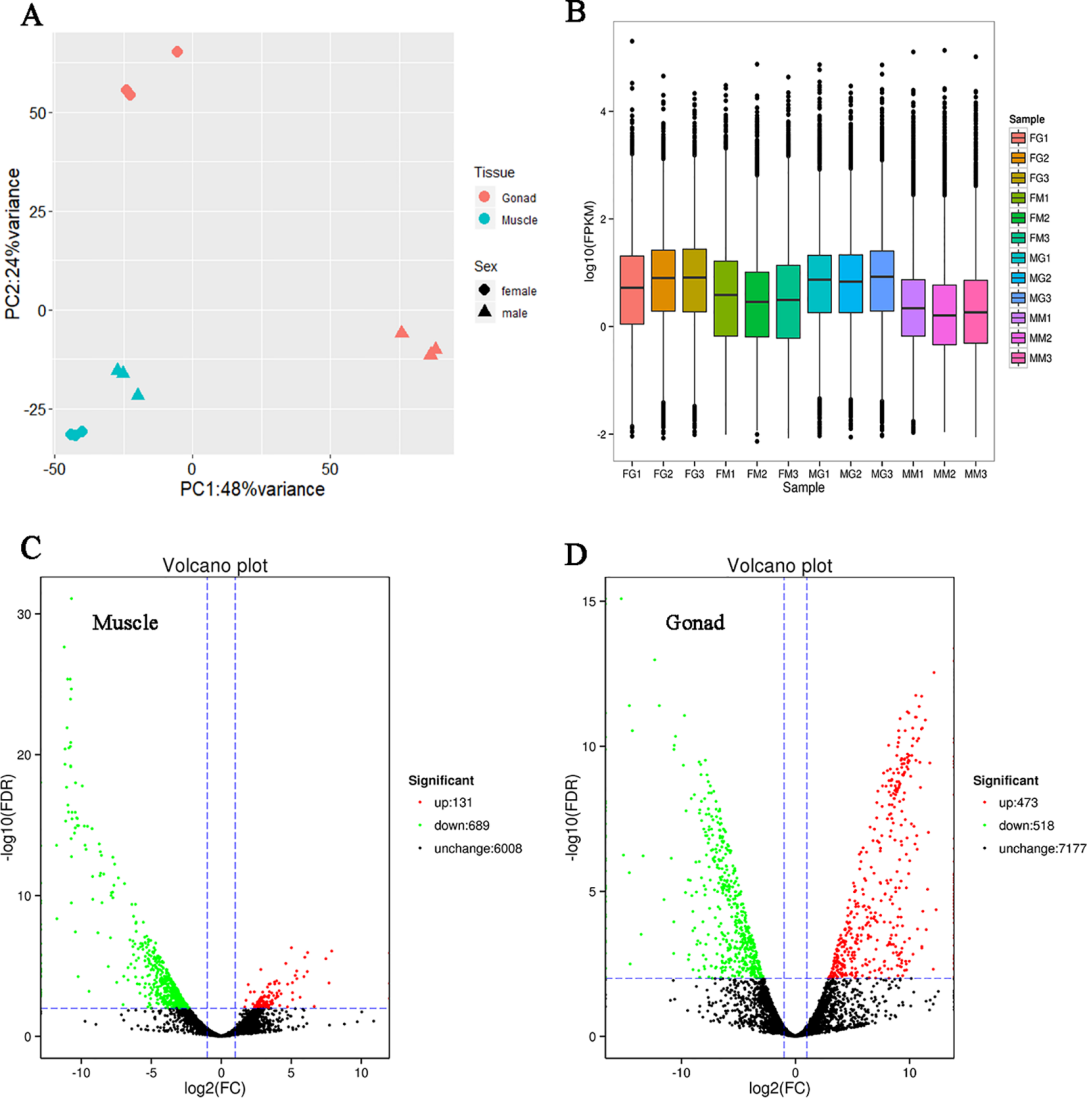
Expression profile reflected by the short reads RNA sequencing data. **(A)** Principal component analysis of the 12 samples. Each point represents one sample, with shape indicating sex, and color indicating tissue. **(B)** Boxplot of the fragments per kilobase of transcript per million fragments mapped (FPKM) distribution of each sample. The abscissa represents different samples. The first letter of the sample name represents sex (the “F” means female, and “M” means male). The second letter of the sample name represents tissue (the “G” means gonad, and “M” means muscle). The number of the sample name represents the different individuals in the same group. The ordinate represents the logarithm of the sample expression FPKM. The graph measures the expression level of each sample from the perspective of the overall dispersion of expression quantity. The last two charts were expression volcano plot of differentially expressed transcripts (DETs) in muscle **(C)** and gonad **(D)**. Each point presents a transcript. The abscissa represents the logarithm of the expression fold change of male relative to female. A larger absolute value indicating a larger expression difference between the male and female. The ordinate represents the negative logarithm of the statistical significance of the expression difference. The larger value indicating the more significant expression difference between male and female, and the better reliability of the screened DETs. The red dots represent up-regulated DETs, the green dots represent down-regulated DETs, and the black dots represent non-DETs.

Twelve samples, including three muscles and three gonads of both male and female shrimps were sequenced. We obtained a total of 88.99 Gb clean data (**Supplementary Table S5**). After mapping with the high-quality non-redundant FL transcripts, expression level of each transcript was quantified. The principal component analysis (PCA) showed that the tissue is the most effected factor for the gene expression, meanwhile the factor of sex played a greater role in gonad than in muscle (**Figure 1**).

The median of fragments per kilobase of transcript per million fragments mapped (FPKM) distribution of expressed transcripts was observed higher in gonad than muscle (**Figure 1**). There were 131 male-biased and 689 female-biased transcripts in muscle (**Figure 1** and **Supplementary Table S6**), while in gonad the number of male-biased transcripts was 473 and female-biased transcripts was 518 (**Figure 1** and **Supplementary Table S7**). This result indicated that in muscle, significantly more transcripts expressed higher in female than male.

For the 162 AS events, we checked their expression in the two tissues. However, no significantly differential expression between different sexes was detected.

### Sex-Biased Expression Transcripts in Muscle Provide Some Clues for Study of Sexual Dimorphism

**Figure 2:**
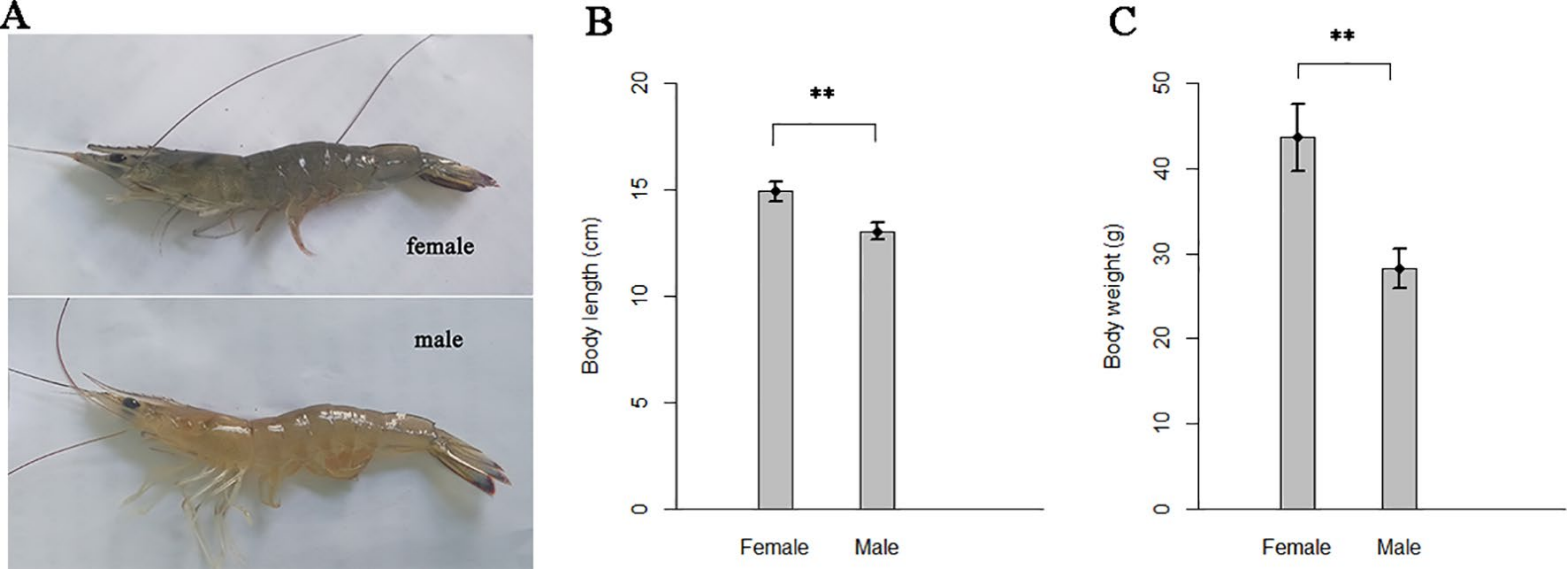
Differences in body color and body size of the two sexes shrimps. **(A)** Body color. **(B)** Body length and **(C)** body weights at 6-months-old. Thirty female and thirty male full-sib shrimps which farmed at a same pond were measured. The line on bar represents standard deviation. “**” indicates significant differences between the two sexes with one-way analysis of variance (P < 0.01).

The body color of adult female shrimps of *F. chinensis* tend to blue, while the males tend to yellow (**Figure 2**). The adult females showed significantly more excellent performance in body length and body weight than males (**Figures 2B, C**). Since the two sexes shared identical genomes except for several potential sex-linked regions (Xie et al., 2008), sexual dimorphism could stem from gene expression differences between sexes.

**Figure 3:**
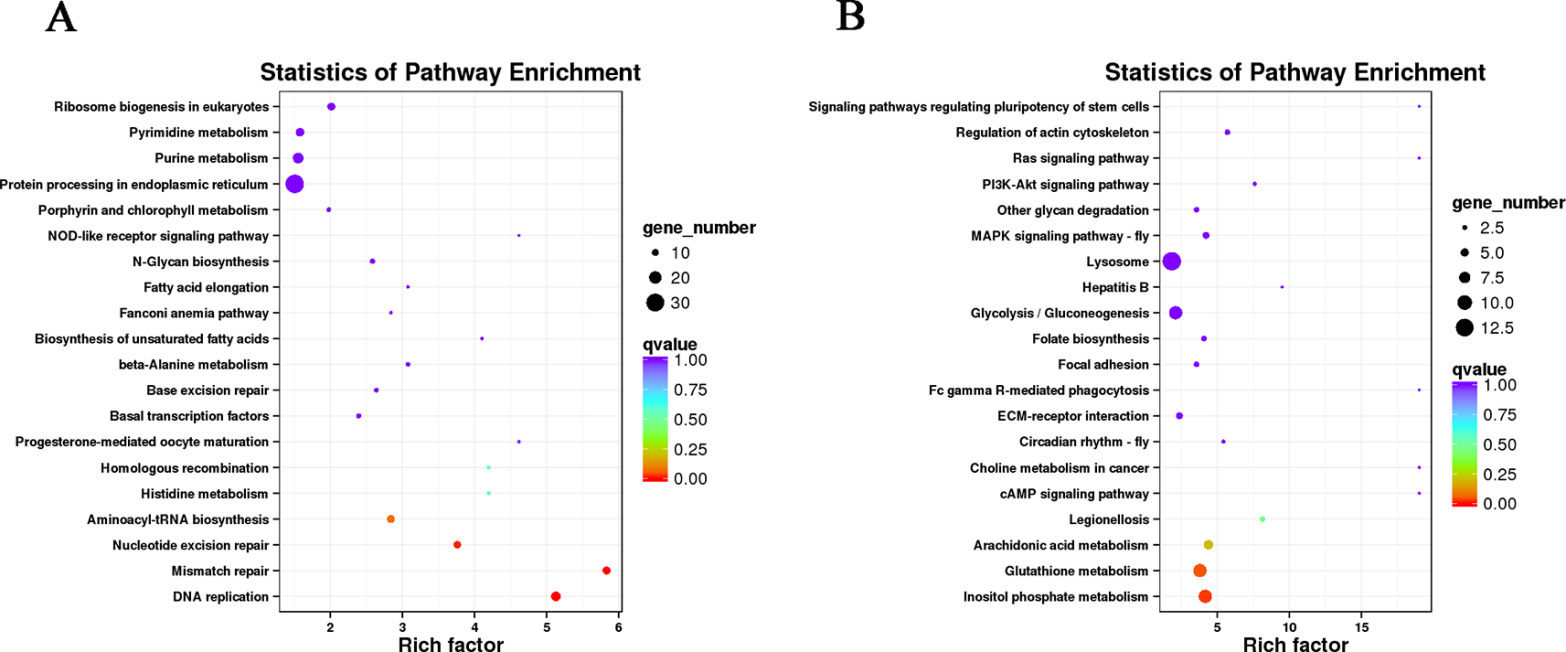
Pathway enrichment of differentially expressed transcripts in muscle **(A)** and gonad **(B)**. Each circle represents a Kyoto Encyclopedia of Genes and Genomes pathway. The vertical axis displays the pathway name and the horizontal axis represents the enrichment factor, which indicating the ratio of the proportion of transcripts annotated into a certain pathway in differentially expressed transcripts (DETs) to the proportion of transcripts annotated into that pathway in all transcripts. A higher enrichment factor represents a more significant enrichment level of DETs in this pathway. The color of the circle represents q-value, which is the P value after correction of multiple hypothesis test. The smaller q-value indicates more reliable enrichment of DET in this pathway. The size of the circle indicates the number of transcripts enriched in the pathway.

The DETs were annotated with Gene Ontology database (**Supplementary Figures S5** and **S6**), and pathway annotation analysis helps to further interpret transcript functions (**Supplementary Figure S7**). In muscle, we observed many DETs participated in pathways related to the genetic information processing, like DNA replication, mismatch repair, nucleotide excision repair, aminoacyl-transfer RNA biosynthesis, Homologous recombination, ribosome biogenesis in eukaryotes and protein processing in endoplasmic reticulum (**Figure 3**). Besides, abundant of DETs involved in the metabolism of substances and energy, like N-glycan biosynthesis, beta-alanine metabolism, histidine metabolism, fatty acid elongation, biosynthesis of unsaturated fatty acids, purine metabolism, and pyrimidine metabolism. We also observed some DETs were enriched into the pathway of porphyrin and chlorophyll metabolism, which may be relevant to the body color of the shrimps. Furthermore, a pathway related to reproduction named progesterone-mediated oocyte maturation was digged out with our result.

**Figure 4:**
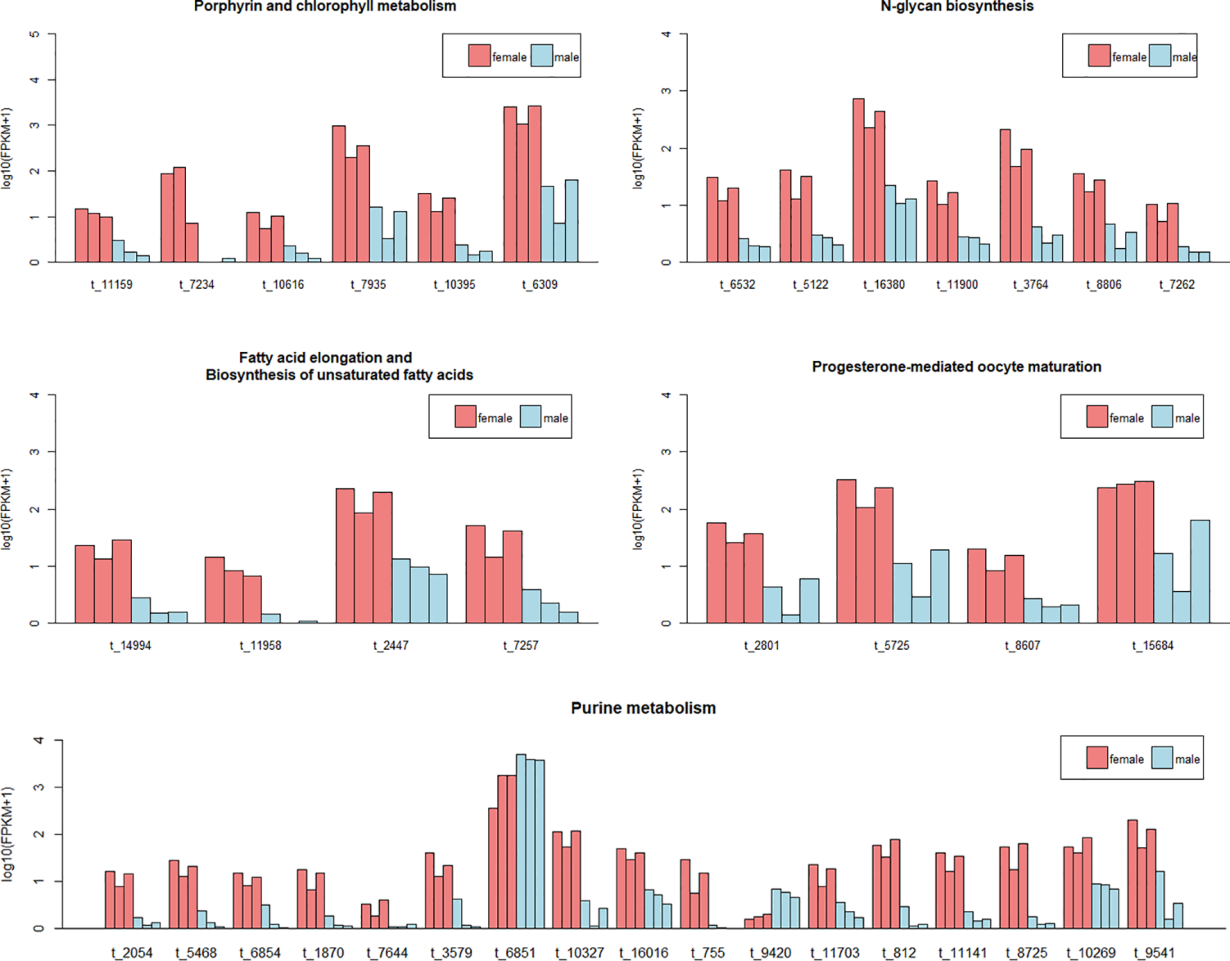
The expression level of the differentially expressed transcripts (DETs) of muscle in five pathways. Each bar represents one sample. Different colors indicate different sexes. The abscissa represents the transcript ID. The ordinate represents the logarithm of the (FPKM+1). Since the DETs in pathways of fatty acid elongation and biosynthesis of unsaturated fatty acids were same, we combined the two pathways in one plot.

We have exacted the expression information of the DETs in these pathways, and found that nearly all these transcripts were female-biased expression (**Figure 4** and **Supplementary Table S8**).

### Ribonucleic Acid Sequencing of Gonad Identified Important Transcripts Action on Reproduction of *Fenneropenaeus chinensis*

The DETs in gonad mostly participated in cellular processes, like focal adhesion, signaling pathways regulating pluripotency of stem cells, and lysosome (**Figure 3**). There were also some substance metabolism process pathways, like arachidonic acid metabolism, glutathione metabolism, inositol phosphate metabolism, folate biosynthesis, glycolysis/gluconeogenesis, and other glycan degradation. Some signal transduction pathways were screened out, such as MAPK signaling pathway-fly, PI3K-Akt signaling pathway, Ras signaling pathway, cyclic adenosine 3,5-monophosphate (cAMP) signaling pathway, and extracellular matrix-receptor interaction. Furthermore, a pathway named circadian rhythm was enriched by several DETs.

**Figure 5:**
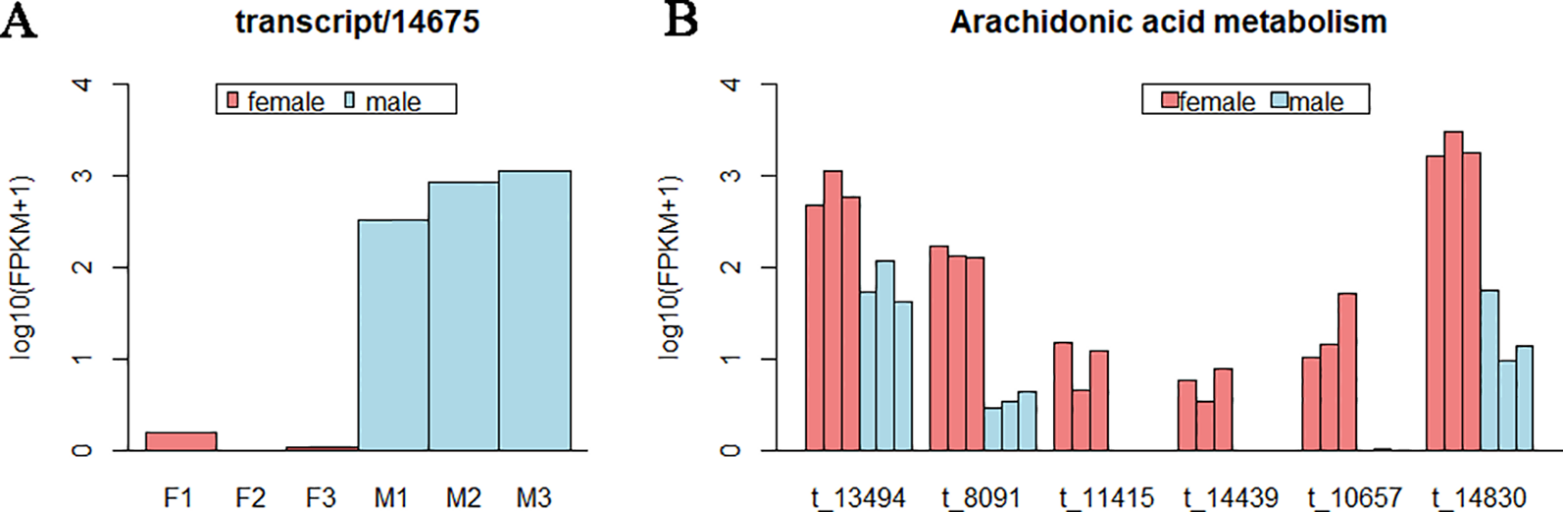
The expression level of some differentially expressed transcripts (DETs) in gonad. **(A)** Expression level of the DET (transcript/14,675) shared by pathways of Fc gamma R-mediated phagocytosis, Ras signaling pathway, cyclic adenosine 3,5-monophosphate signaling pathway, and choline metabolism in cancer. **(B)** Expression level of DETs in the pathway of arachidonic acid metabolism. Each bar represents one sample. Different colors indicate different sexes. The abscissa represents the transcript ID. The ordinate represents the logarithm of the (FPKM+1).

Interestingly, for the pathways of Fc gamma R-mediated phagocytosis, Ras signaling pathway, cAMP signaling pathway, and choline metabolism in cancer, they shared one same DET (transcript/14,675), and only this DET enriched in these pathways. This transcript expressed in males far beyond females (**Figure 5** and **Supplementary Table S9**), which action on lipid transport and metabolism. In gonad, DETs in most of pathways were female-biased or male-biased expression irregularly, while in the pathway of arachidonic acid metabolism, all the six DETs were female-biased (**Figure 5**).

## Discussion

In this study, we obtained 10,795 high-quality FL transcripts, which was relatively less than the similar work on *L. vannamei* (Zhang et al., 2019). Besides the species difference, it could be attribute to the strict parameters setting in the process of FL transcript cluster. In order to obtain high-quality consensus isoforms, there is possibility that multi-copy sequences of a same transcript were divided into different clusters, which inevitably resulting in redundant sequences. At the same time, degradation of the 5’ end during the sequencing also could result in different copies of a same transcript being divided into different clusters. Considering about that, we clustered the FL transcripts with a strict condition. Completeness assessment of the FL transcriptome (**Supplementary Figure S2**) proved the reliability of our result.

The median of the FPKM distribution of expressed transcripts in gonad was higher than in muscle. This reflected a higher expression abundance of transcripts in gonad to a certain extent. It also could attribute to limited genes expressing in gonad. Moreover, since the FPKM value was to normalize the expression level by eliminating the effect of the sequencing depth and gene length, more longer transcript expressed in muscle could cause this result in FPKM distribute (Wagner et al., 2012).

Some aspects of sexual dimorphism result from genes located on the sex chromosomes (Rice, 1984). For *F. chinensis*, the sex determination and differentiation mechanism have yet to be elucidated. It remains unclear whether the ZW sex determination system (Xie et al., 2008) or some more complicated mechanism instead of simple XY or ZW system (Li et al., 2003) existing in the sex determination of *F. chinensis* (Li et al., 2012). However, many organisms lacking of sex chromosomes entirely also performed pronounced dimorphisms, and even though some species possess sex chromosomes, a majority of dimorphism controlled by genes presenting in both sexes (Bachtrog et al., 2014). Expression is one way that genes can be deployed differently. Genes show differential expression between male and female are referred to as sex-biased genes (Mank, 2017). The magnitude of sex-biased expression amplifies along with development, and reach the most manifest in adults (Mank et al., 2010; Perry et al., 2014). Therefore, we collected samples at 5-months old in this study, when they approaching the mating stage, expecting to capture more sex-biased expression genes.

The *F. chinensis* is an annual shrimp. Female shrimps migrate to warmer sea area to overwinter after mating, and swim back to original coast for oviposition. In the meantime, selective pressure forced on females. The female shrimps should store enough energy for the migration and preparation for oviposition, which could result in the bigger body size.

There were significantly more transcripts showed female-biased expression in muscle, which was speculated attributing to the better growth performance of females. With the DETs in muscle, some potential pathways relating to the sexual dimorphism of *F. chinensis* were unearthed. There were several pathways involved in metabolism of substances and energy. The N-glycan biosynthesis, beta-alanine metabolism, and histidine metabolism participate in protein synthesis. The fatty acid elongation and biosynthesis of unsaturated fatty acids related to fat synthesis. The purine metabolism takes part in the energy supply of organisms (Nisr and Affourtit, 2016). The metabolism of substances and energy was considered relevant to the growth rate (Krieger, 1978; Vahl, 1984). Since most of transcripts in these pathways were female-biased, it supported the conjecture that these DETs were responsible for the fast growth of females. The fast growth always accompanied by frequent cell division and gene expression (Dayton and White, 2008), which reflected in the enrichment of DETs in the pathways of genetic information processing.

There was scarcely any research study the body color of *F. chinensis*. In this research, we caught a pathway possibly related to the pigmentation, named porphyrin and chlorophyll metabolism. Porphyrins and their derivatives are widely found in important organelles related to energy transfer in organisms (Milne et al., 2015; Galvan et al., 2016). The porphyrins show different colors when they coordinate with different metal ions. It is mainly found in heme (iron porphyrin) and hemocyanin (copper porphyrin) in animals, vitamin B12 (cobalt porphyrin), and chlorophyll (magnesium porphyrin) in plants. Interestingly, the content of a porphyrin derivative, protoporphyrin IX, was reported responsible for the brown color depth of eggshell in chicken (With, 1974). Considering about that, we proposed three hypotheses about the color difference between the two sexes: I. The hemocyanin content effect the body color; II. Other porphyrin derivatives deposited in the muscle or epidermis of female resulting in the deeper color; III. Other substances have no connection with porphyrin play roles. However, we cannot make it clear based on the current research. Further verification experiments are required to answer this question.

Although the muscle is not the reproductive tissue, there still some reproduction-related genes expressed. The pathway of progesterone-mediated maturation was digged out by the DETs in muscle. We sampled the shrimps at 5-months-old, when they approaching sexual maturity, and about to ready for the mating a month later. It is a key stage of reproduction for *F. chinensis.* The four DETs in this pathway were all female-biased, indicating an active oocyte development in female.

Unlike the muscle, DETs in gonad were irregularly female- or male-biased expression in most of pathways. Exceptionally, all the six DETs in the pathway of arachidonic acid metabolism were female-biased. The arachidonic acid (AA) is one of the initiators in prostaglandin biosynthesis (Khanapure et al., 2007), and prostaglandin could regulate reproductive function of female (Villars et al., 1985; Norberg et al., 2017). The AA was reported being largely incorporated into ovarian lipids exceeding other fatty acids (Johnson et al., 2017). It is the fatty acid precursor of an important signal molecule for crustacean reproduction (Kangpanich et al., 2016). Our result revealed that the AA was critical for the reproduction of females of *F. chinensis* at the pre-breeding stage. An appropriate feed proportion with adequate AA at this stage may be benefit for the reproduction of *F. chinensis*.

There was a transcript (transcript/14,675) expressed in male far beyond female, and participated in several important signaling pathways. This transcript was predicted to encode phospholipase D alpha 1-like, which could function on lipid transport and metabolism. The phospholipase D has been reported to play important roles on reproduction of males in other species (Vinggaard and Hansen, 1993; Lee et al., 2011; Zhang et al., 2018). This result provides a clue for research on the function of this protein in reproduction of male shrimps.

A pathway named circadian rhythm was enriched. We have known the existence of endogenous rhythms in crustacean to cope with the effect of tide, light, salinity, and so on (Naylor, 1985). The *F. chinensis* mate at a fixed time of each year, and then migrate to the south warmer area to overwinter (Kong et al., 2010). This series of behaviors were closely linked with circadian rhythm. The two DETs in this pathway was predicted to be transcribed from gene *vrille*, which was reported to drive rhythmic behavior in *Drosophila* (Gunawardhana and Hardin, 2017). We speculated that the gene *vrille* also played important role in rhythm behavior of *F. chinensis*.

In conclusion, our study profiled the transcriptome of *F. chinensis.* We further identified the DETs between two sexes which potentially responsible for the sex dimorphism in *F. chinensis*, such as growth, body color, and some reproductive-related functions. However, further researches were needed to verify the current preliminary result. Our results provided a basis for understanding the underlying molecular mechanism of sexual dimorphism in *F. chinensis*.

## Materials and Methods

### Sample Collection and Handling

We picked two male and two female shrimps of “Huanghai No. 1” randomly at 5-months-old. Muscle, gonad, hepatopancreas, intestine, ganglion, heart, and sputum were collected and frozen in liquid nitrogen. Total RNA was extracted using TRIzol (Invitrogen, USA) with the standard protocols from the manufacturer. The RNA quality was assessed by NanoDrop 2000 spectrophotometer (Thermo Fisher Scientific Inc.) and agarose gel electrophoresis (AGE). Twenty-eight RNA samples (4 individuals × 7 tissues) were mixed into one pool with equal amount of nucleic acids. The mixed pool was applied to single-molecule FL transcriptome sequencing.

Another 15 female and 15 male shrimps were chosen for Illumina-based RNA-seq. We collected their gonad (female: ovary, male: testis) and muscle. Total RNA was extracted as stated above. Each five RNA samples of same sex and same tissue were mixed into one pool. The three pools of each sex were treated as biological duplicates.

To measure the body weight and body length of *F. chinensis*, we picked 30 female and 30 male full-sib shrimps of “Huanghai No. 1” randomly at 6-months-old, which were farmed at a same pond. The shrimps were measured with living body. Body length refers to the length from the base of the eyestalk to the end of the tail, when the shrimp measured as straight as possible.

### Library Construction and Sequencing

We constructed FL transcriptome sequencing library of 1–6 kb complementary (cDNA) for the mixed pool sample. The library was sequenced on one SMRT Cell of Pacific Biosciences (PacBio) platform. Brieﬂy, SMARTer™ PCR cDNA Synthesis Kit (Pacific Biosciences, Menlo Park, CA, USA) was used to generate first- and second-strand cDNA from mRNA. After a round of polymerase chain reaction (PCR) amplification and end repair, SMRTbell™ hairpin adapters were ligated. By exonuclease digestion, we obtained a 1–6 kb cDNA library.

Twelve libraries of two tissues and two sexes (three duplicates) were constructed following the protocol of the Gene Expression Sample Prep Kit (Illumina, San Diego, CA, USA). The libraries were sequenced by Illumina NovaSeq S4 platform with paired-end (PE) 150 nt.

### PacBio Long Read Processing

Raw reads were processed into error corrected reads of insert (ROIs) using Iso-Seq pipeline (Pacific Biosciences, Menlo Park, CA, USA) (Rhoads and Au, 2015) with minFullPass = 1 and minPredictedAccuracy = 0.90. FL, non-chimeric (FLNC) transcripts were determined by searching for the polyA tail signal and the 5’ and 3’ cDNA primers in ROIs. We used ICE (iterative clustering for error correction) to obtain FL consensus isoforms and they were further polished. Then the high-quality FL consensus isoforms were classified with the criteria post-correction accuracy above 99%. The low-quality FL consensus transcripts were corrected by our Illumina short reads RNA-seq data using the proovread software (Hackl et al., 2014), and merged with the high-quality FL consensus transcripts. Then the merged high-quality FL transcripts were removed redundancy using cd-hit (Li and Godzik, 2006) (identity > 0.99). Gene function was annotated by BLAST (Altschul et al., 1997) (version 2.2.26) based on the following databases: NR (NCBI non-redundant protein sequences) (Pruitt et al., 2007); Pfam (protein family) (Finn et al., 2014); KOG/COG/eggNOG (clusters of orthologous groups of proteins) (Tatusov et al., 2000; Koonin et al., 2004; Jensen et al., 2008); Swiss-Prot (a manually annotated and reviewed protein sequence database) (The UniProt, Consortium, 2017); KEGG (Kyoto Encyclopedia of Genes and Genomes) (Kanehisa et al., 2004); GO (Gene Ontology) (Ashburner et al., 2000).

The structure analysis of the transcriptome was as follows:

#### Alternative Splice

We used Iso-Seq^™^ data directly to run all-*vs.*-all BLAST with high identity settings, BLAST alignments that met all criteria were considered products of candidate AS events: there should be two HSPs (high-scoring segment pair) larger than 1,000 bp in the alignment; the two HSPs have same forward/reverse direction, within the same alignment; one sequence should be continuous, or with a small “overlap” size (smaller than 5 bp), the other one should be distinct to show an “AS gap”; the continuous sequence should pretty much completely align to the distinct sequence; the AS Gap should larger than 100 bp and at least 100 bp away from the 3’/5’ end.

#### Simple Sequence Repeat Detection

Simple sequence repeats (SSRs) of the transcriptome were identified using MISA (http://pgrc.ipk-gatersleben.de/misa/).

#### Coding Sequence Detection

Candidate coding regions within transcript sequences were identified by TransDecoder (https://github.com/TransDecoder/TransDecoder/releases) (version 5.5.0). We used the following criteria: 1) a minimum length open reading frame (ORF) is found in a transcript sequence; 2) a log-likelihood score similar to what is computed by the GeneID software (http://genome.crg.es/software/geneid/) is > 0; 3) the above coding score is greatest when the ORF is scored in the 1st reading frame as compared to scores in the other five reading frames; 4) if a candidate ORF is found fully encapsulated by the coordinates of another candidate ORF, the longer one is reported. However, a single transcript can report multiple ORFs (allowing for operons, chimeras, etc); 5) optional the putative peptide has a match to a Pfam domain above the noise cutoff score.

#### Long Non-Coding Ribonucleic Acid Analysis

Four computational approaches include coding potential calculator (CPC) (Kong et al., 2007), Coding-Non-Coding Index (CNCI) (Sun et al., 2013), Coding Potential Assessment Tool (CPAT) (Wang et al., 2013), and Pfam database (Finn et al., 2014) were combined to sort non-protein coding RNA candidates from putative protein-coding RNAs in the transcripts. Putative protein-coding RNAs were filtered out using a minimum length and exon number threshold. Transcripts with length more than 200 nt and possess more than two exons were selected as lncRNA candidates and further screened using CPC/CNCI/CPAT/Pfam that have the power to distinguish the protein-coding genes from the non-coding genes.

### Illumine-Based Ribonucleic Acid Sequencing Data Processing

Raw reads of FASTQ format were firstly processed through in-house Perl scripts. Briefly, clean reads were obtained by removing reads containing adapter or ploy-N and low-quality reads from raw data. At the same time, Q20, Q30, GC-content, and sequence duplication level of the clean data were calculated. All the downstream analyses were based on clean data with high quality.

The clean reads of each RNA-seq library were aligned to the FL reference transcriptome to obtain unique mapped reads by using the tool of STAR (Dobin et al., 2013) (version 2.5.0b) with default parameters. Only reads with a perfect match or one mismatch were further analyzed and annotated based on the reference transcriptome. The read counts were adjusted by edgeR program package (Robinson et al., 2010) (version 3.22.0). Expression level of each transcript for each tissue was calculated and normalized into FPKM values by RSEM software (Li and Dewey, 2011) (version 1.2.19). The resulting FDR (false discovery rate) was adjusted using the PPDE (posterior probability of being DE) method in EBSeq package (Leng et al., 2013) (version 1.24.0). We set the conditions of FDR < 0.05 and |log2(foldchange)|≥1 as the threshold for significantly differential expression.

### Functional Enrichment Analysis of DETs

GO enrichment analysis of the DETs was implemented by the R packages of GOseq (Young et al., 2010) (version 1.34.1) based on the Wallenius non-central hyper-geometric distribution, which can adjust for gene length bias in differential expression genes (DEGs). All of the transcripts of *F. chinensis* annotated in this study were used as the background data.

KEGG (Kanehisa et al., 2017) is a database resource for understanding high-level functions and utilities of the biological system (http://www.genome.jp/kegg/). We used KOBAS software (Mao et al., 2005) (version 3.0.0) to test the statistical enrichment of DETs in KEGG pathways.

## Data Availability Statement

The sample information was registered as BioProject with accession number PRJNA558194 and BioSample with accession number from SAMN12429759 to SAMN12429771. Raw sequence generated by SMRT and Illumina platform was deposited into the NCBI Sequence Read Archive (SRA) with accession number SRR9894260.

## Ethics Statement

The animal study was reviewed and approved by The Animal Care and Use Committee of the Yellow Sea Fisheries Research Institute, Chinese Academy of Fishery Sciences.

## Author Contributions

QW analyzed and interpreted the sequencing data and drafted the manuscript. YH participated in collecting the samples and improved the manuscript. JL conceived of the study, and participated in its design and coordination. All authors read and approved the final manuscript.

## Funding

This work was supported by the China Agriculture Research System (Grant No. CARS-48), grants from the Program of Taishan Industrial Experts (Grant No. LNJY2015002), National Natural Science Foundation of China (Grant No. 31902367) and China Postdoctoral Science Foundation (Grant No. 2018M642730).

## Conflict of Interest

The authors declare that the research was conducted in the absence of any commercial or financial relationships that could be construed as a potential conflict of interest.
