# Supplementary material for: Conjoint Analysis of SMRT- and Illumina-Based RNA-Sequencing Data of Fenneropenaeus chinensis Provides Insight Into Sex-Biased Expression Genes Involved in Sexual Dimorphism
Source: Front Genet. 2019 Nov 15;10:1175. doi: 10.3389/fgene.2019.01175 (PMC6872642; doi:10.3389/fgene.2019.01175)
Supplement: Supplementary file 2 [file DataSheet_2.docx]

**Supplementary figures:**


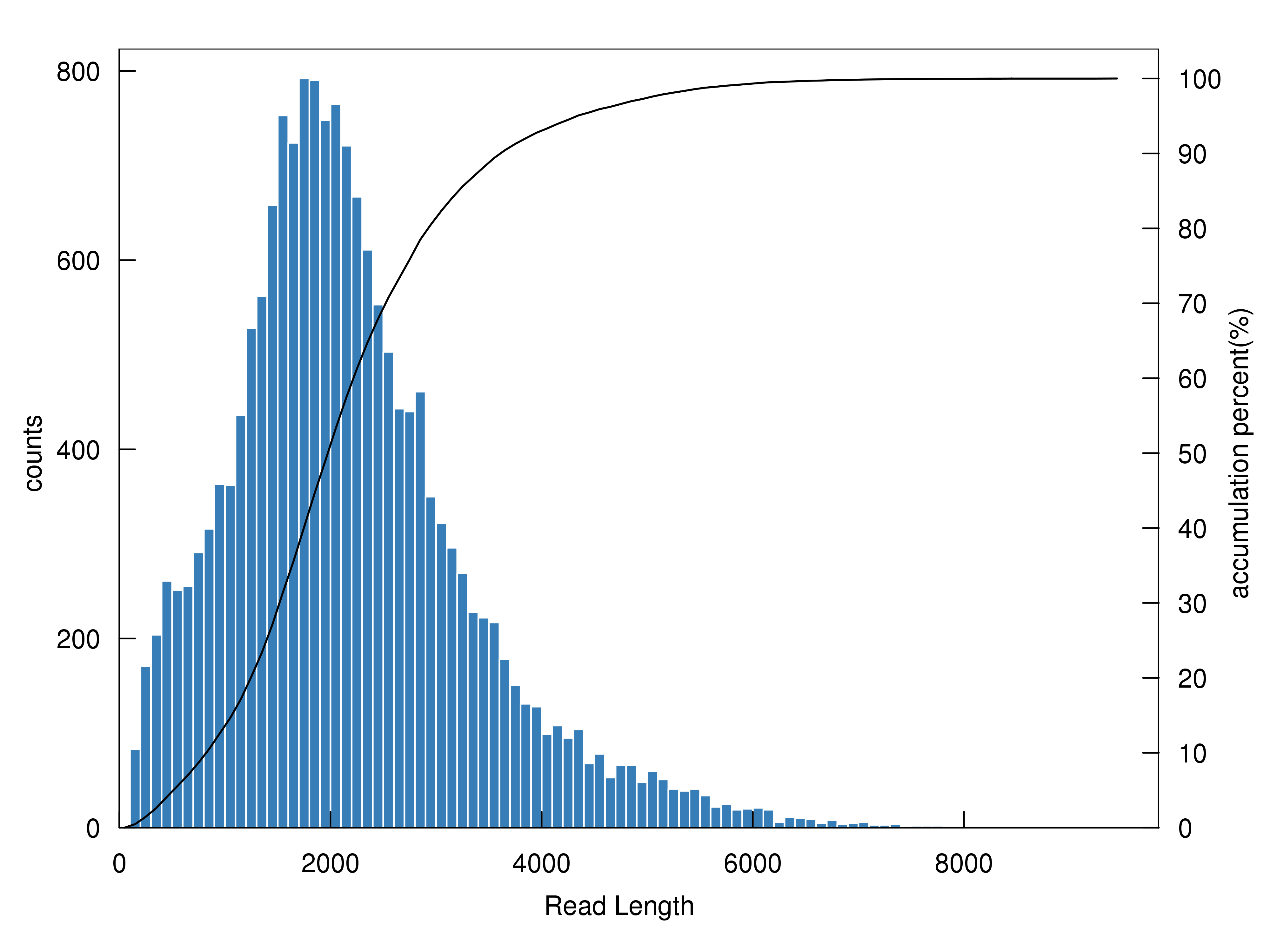
Figure S1 Consensus isoforms read length distribution. The abscissa refers to the consensus isoform sequence length distribution. The left ordinate refers to consensus isoform sequence length frequency distribution histogram. The right ordinate refers to consensus isoform sequence length cumulative frequency curve.


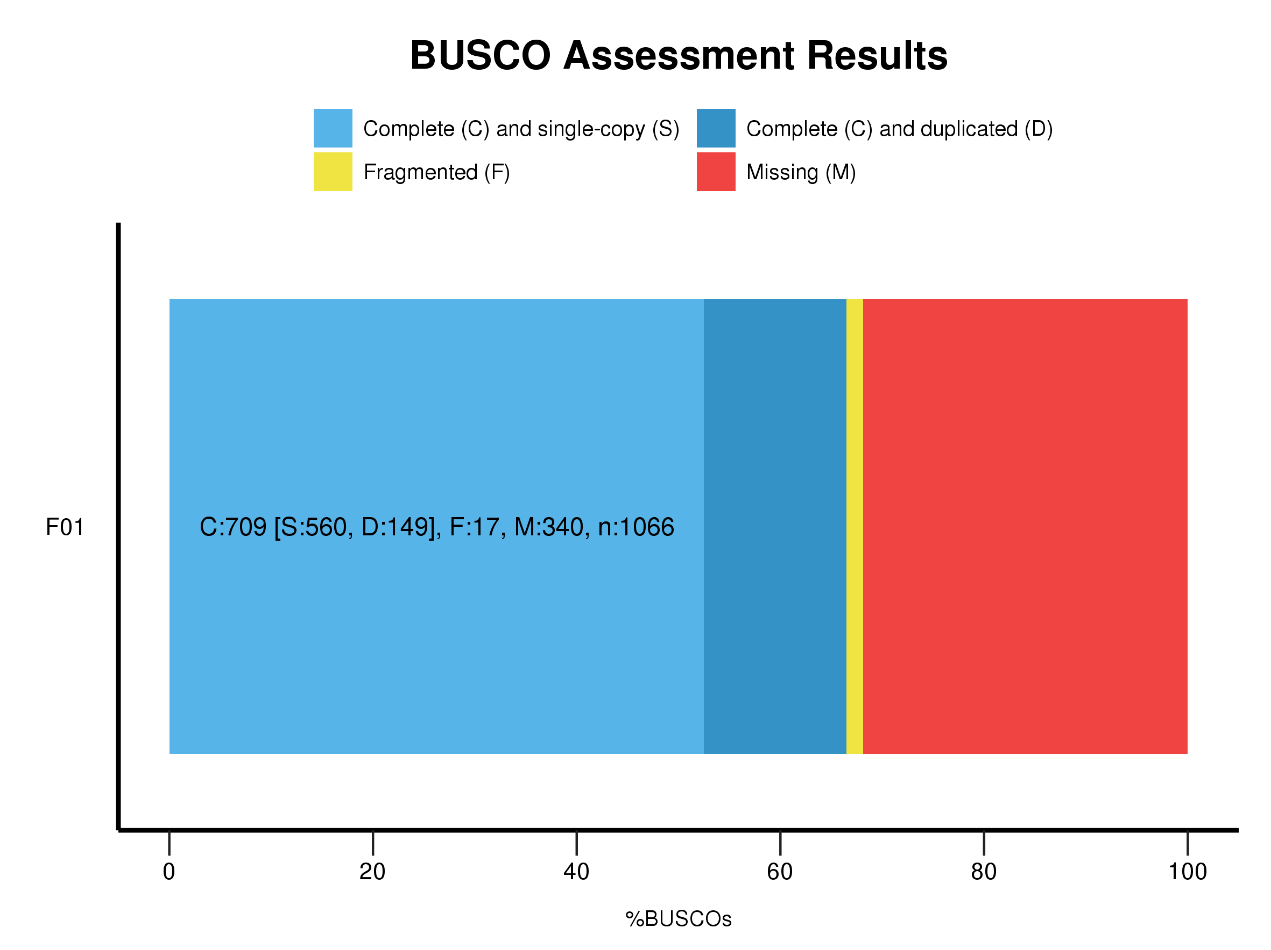
Figure S2 Completeness assessment of the non-redundant FL transcriptome


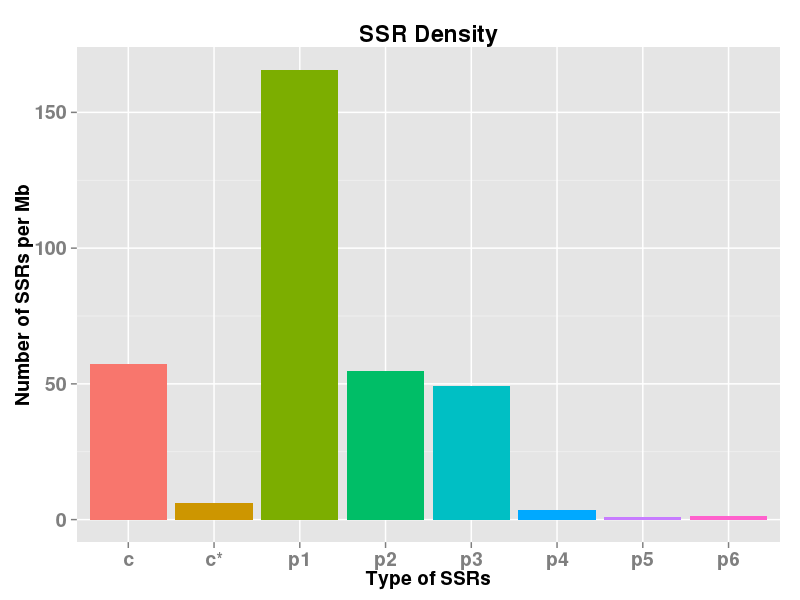
Figure S3 Density distribution statistics of different types of SSR. The “c” refers to sequence containing more than one SSR. “c*” refers to sequence containing more than one SSR but difficult to distinguish adjacent two SSR. “p1” refer to Mono-nucleotide repeat SSR. “p2” refer to Di-nucleotide repeat SSR. “p3” refer to Tri-nucleotide repeat SSR. “p4” refer to Tetra-nucleotide repeat SSR. “p5” refer to Penta-nucleotide repeat SSR. “p1” refer to Hexa-nucleotide repeat SSR.


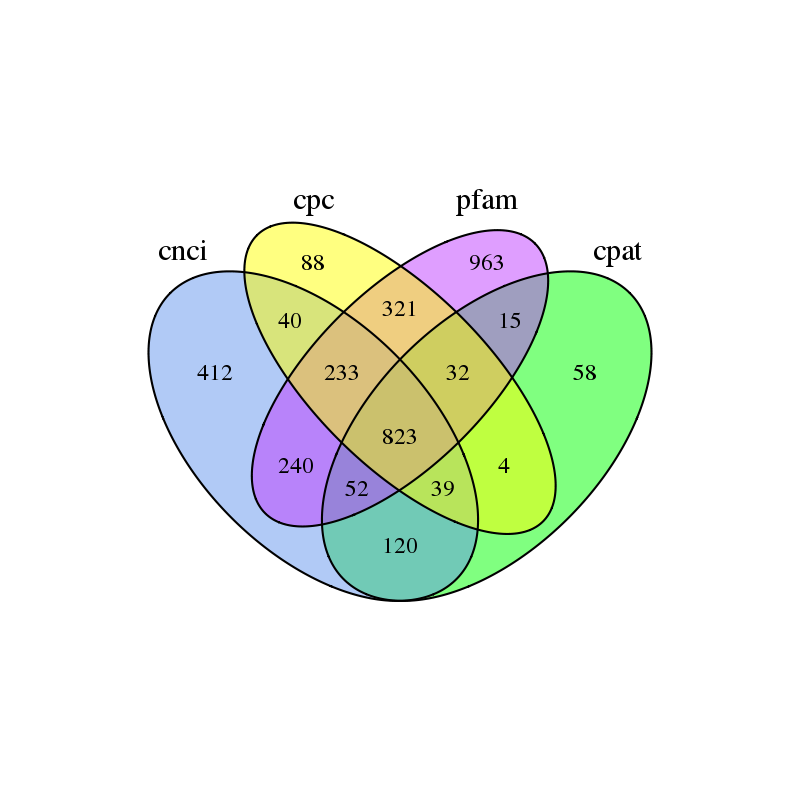


Figure S4 Venn diagram of noncoding transcripts identification using four method.


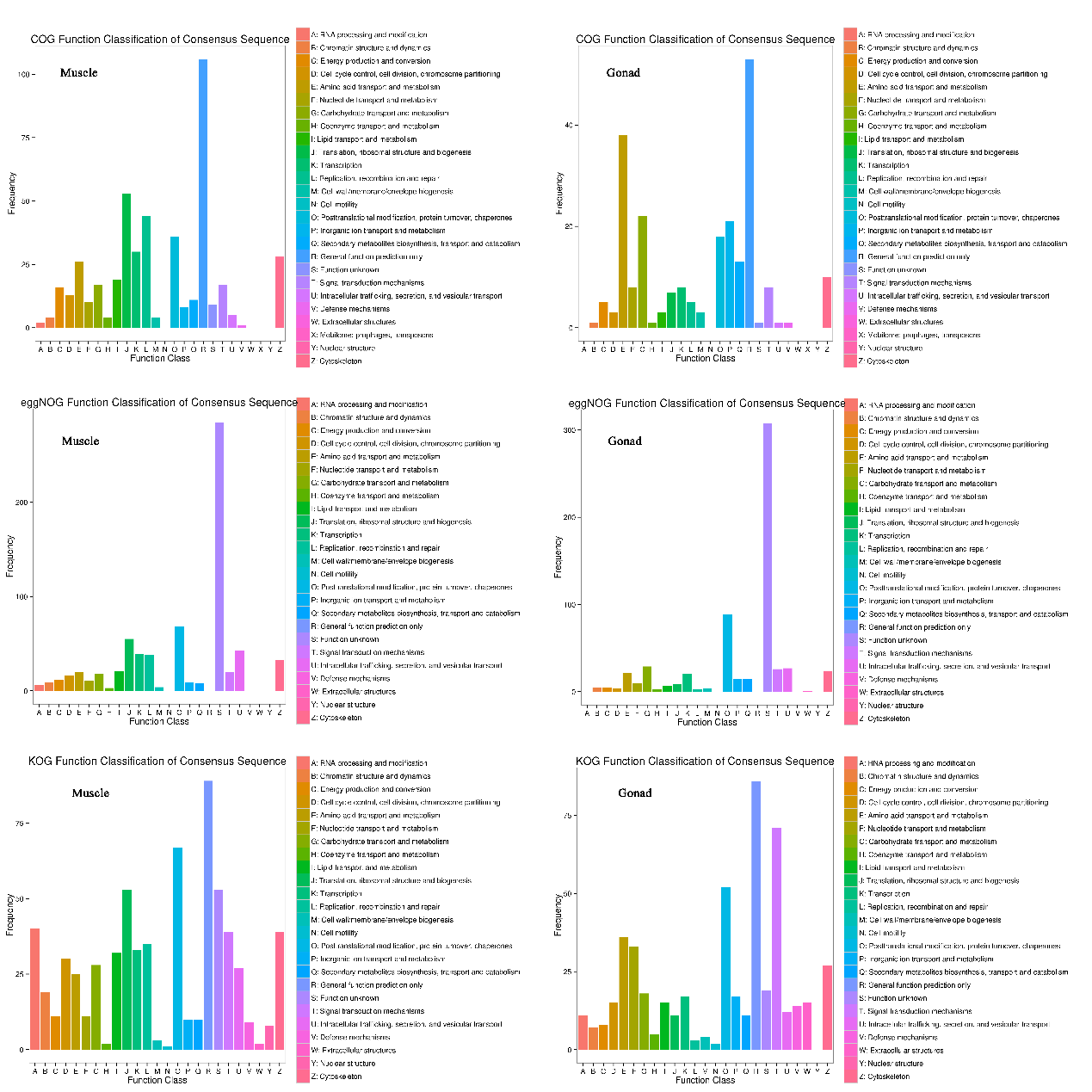
Figure S5 Statistics of DETs COG/eggNOG/KOG annotation classification.


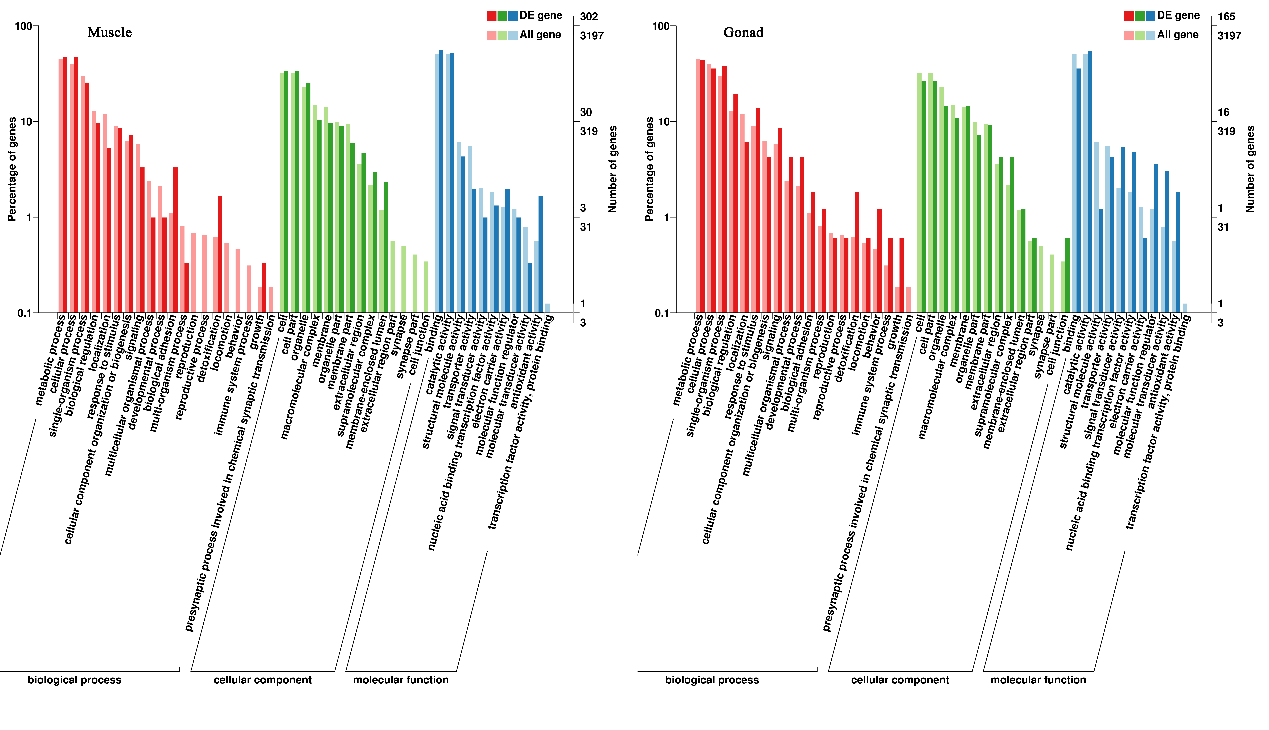
Figure S6 Statistics of DETs GO annotation classification.


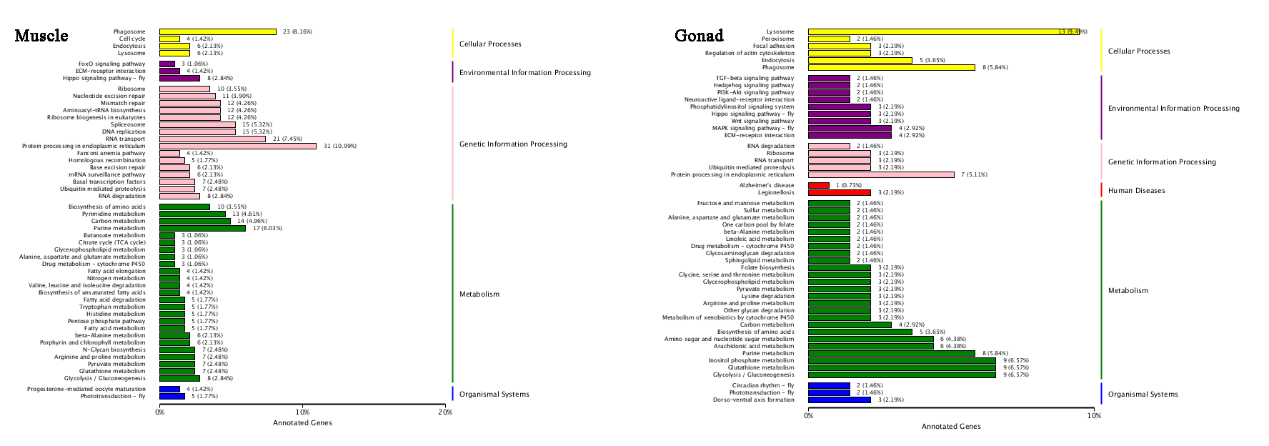
Figure S7 KEGG pathway classification of DETs.
